# Serum vitamin D and diabetic foot complications

**DOI:** 10.1080/2000625X.2019.1579631

**Published:** 2019-02-19

**Authors:** Robert M. Greenhagen, Robert G. Frykberg, Dane K. Wukich

**Affiliations:** a Midwest Foot and Ankle Fellowship, Foot and Ankle Center of Nebraska, Omaha, NE, USA; b Midwestern University Program in Podiatric Medicine, Midwestern University, Fountain Hills, AZ, USA; c Department of Orthopaedic Surgery, UT Southwestern Medical Center, Dallas, TX, USA

**Keywords:** Charcot, neuroarthropathy (CN), diabetic peripheral neuropathy (DPN), diabetic foot infection (DFI), diabetic foot ulcer (DFU), peripheral arterial disease (PAD), vitamin D

## Abstract

**Background**: Foot complications such as ulceration and neuropathy are common complications of diabetes mellitus (DM). Previous reports have demonstrated a possible increased risk of these complications in diabetic patients with low levels of serum vitamin D.**Objectctive**: The purpose of this study is to compare serum vitamin D levels in diabetic patients with and without Charcot neuroarthropathy (CN), peripheral arterial disease (PAD), infection (DFI), ulceration (DFU), and peripheral neuropathy (DPN).

**Design**: A retrospective chart review of all patients undergoing foot and ankle surgery with a history of DM over a 13 month period was performed. From this cohort, fifty subjects with CN were matched with 50 without CN and preoperative lab values were compared. A secondary evaluation was performed on the subjects’ other comorbidities including PAD, DFI, DFU, and DPN.

**Results**: Seventy-eight percent of our patients had vitamin D deficiency or insufficiency. Preoperative serum vitamin D levels were not significantly different between diabetic patients with and without CN (p = 0.55). Diabetic patients with PAD (p = 0.03), DFI (p = 0.0006), and DFU (p = 0.04) were all found to have significantly lower serum vitamin D levels than diabetic patients without these complications. Lower levels of serum albumin and higher serum creatinine were also noted with subjects with PAD, DFI, DPN, and DFU. While seasonal serum vitamin D level fluctuation was noted, this difference did not reach statistical significance with the numbers available.

**Conclusion**: We found various lower extremity complications to be associated with low serum vitamin D including PAD, DFI, and DFU. While other studies have questioned the role of vitamin D and CN, we were unable to identify any significant difference between diabetic patients with and without Charcot neuroarthropathy.

**Level of evidence**: Level 2

## Introduction

Serum vitamin D and its role in diabetes-related complications have become an important topic among researchers and physicians in recent years. Normal levels of serum vitamin D levels have been shown to positively affect a number of diseases such as cancer, heart disease, diabetes, hypertension, autoimmune diseases, and insufficiency fractures [1]. All human cells have vitamin D receptors; the role and effect the receptor plays is cell dependent. In endothelial cells, Vitamin D is associated with structural and functional vascular changes, modulated by NADPH oxidase, nitric oxide, and extracellular matrix components [2]. In the pancreas, vitamin D modifies the function of β-cells through several pathways. These include the direct stimulation of insulin secretion through the presence of vitamin D receptors (VDRs) in β-cells [3] and their expression of 1-α-hydroxylase enzyme [4]. It is clear that vitamin D is more than just a simple vitamin. Research has shown that vitamin D is most likely the oldest hormone; it has been used by organisms like phytoplankton dating back to 750 million years ago [5].

Vitamin D deficiency is a serious and far-reaching problem. It has been estimated that one billion people are deficient worldwide [6]. This is most likely secondary to lifestyle habits and the latitude of most major cities. Nursing home patients, elderly, hospital inpatients, pregnant and breastfeeding mothers are at highest risk for deficiency [6]. Studies have raised concern that many patients with diabetes mellitus (DM) may have an underlying vitamin D deficiency [7] and there may be an association with lower extremity complications including Charcot neuroarthropathy [8], arterial disease [9–11], chronic inflammation [12,13], and foot ulcerations [14]. The purpose of this study is to compare serum vitamin D levels in diabetic patients with and without Charcot neuroarthropathy (CN), peripheral arterial disease (PAD), infection (DFI), peripheral neuropathy (DPN), and ulceration (DFU).

## Material and methods

Institutional review board approval was obtained prior to beginning this study. A retrospective review of charts was completed through the department’s comprehensive foot and ankle registry. Our study included any patient who was 18 years or older who underwent foot and ankle surgery by a single surgeon (DKW). All patients were enrolled during their preoperative evaluation with the attending surgeon. During a consecutive 13-month period, 256 surgeries were performed on 160 diabetic patients. For purposes of analysis, only data collected from the patient’s first procedure was used, unless the record was incomplete (such as an emergent surgery). In these cases, the earliest procedure with a complete record was used.

Prior to surgery, patients were screened for peripheral neuropathy and PAD. The following data were included for each subject: age, gender, presence or absence of DM, type of DM (type 1 or 2), presence of comorbidities such as PAD, DPN, tobacco use, history of organ transplantation, and history of and/or current DFU. Preoperative serum glucose, creatinine, glycohemoglobin (HgA1C), albumin, and 25-OH vitamin D3 were recorded. Based on current literature, vitamin D deficiency is defined as a vitamin D3 below 20 ng/mL, and vitamin D insufficiency as a vitamin D3 of 21–29 ng/mL [15]. Fifty diabetic patients with a history of CN were identified and compared to 50 diabetic patients without CN. The non-Charcot subjects were matched to the CN group based on age, gender, and type of DM (type 1 vs. type 2). Secondary comparisons were made within the 100 patients using the variables of PAD, DFI, DPN, and current DFU.

As a routine part of our academic practice, all patients had both feet examined for neurovascular abnormalities. Patients were diagnosed to have DPN using the Michigan Neuropathy Screening Index (MNSI) [16]. This validated instrument relies on Semmes–Weinstein monofilament, vibration testing with the 128-Hz tuning fork, assessment of Achilles reflex, presence or absence of ulceration, and the presence or absence of deformity (claw toes, Charcot’s neuroarthropathy [CN]). In the validation study, diabetic neuropathy was confirmed in patients by a quantitative neurological examination coupled with nerve conduction studies [17]. The cutaneous sensation was assessed with a 5.07 Semmes–Weinstein monofilament using four plantar sites (the first and fifth metatarsal heads, plantar hallux, and heel). These four sites represented a slight modification from the original description of the MNSI. Patients who could sense all four sites with their eyes closed received a score of zero for each foot. Patients who were unable to sense the monofilament in one of the four sites received a score of 0.5 per foot. Patients who were unable to sense two or more of the four sites received a score of 1.0 per foot. Vibratory sensation was evaluated with the 128-Hz tuning fork at the dorsal hallux. If the patients could feel the vibration consistent with the duration that the examiner felt while holding the tuning fork, a score of 0 was given for each foot. Those patients who initially felt the vibration but were unable to sense it after 5 s of dampening received a score of 0.5 for each foot. Those patients who were unable to sense the tuning fork at all or noted absence within 5 s were given a score of 1.0 for each foot. Achilles reflexes were evaluated in a standard musculoskeletal manner [18,19]. An intact reflex without re-enforcement was scored as zero; a reflex that was present with re-enforcement was scored as 0.5 and absent reflex as 1.0. If a foot ulcer was present, a score of 1.0 was given and a zero was scored if no ulcer was present. For the purposes of this study, we defined neuropathic deformity as multiple claw toes in both feet (in association with neuropathy) and/or the presence of CN. Claw toes and CN were evaluated both clinically and radiographically. The presence of a deformity was scored as 1.0 and the absence of deformity as zero. The maximum score per foot was five and the combined maximum total score of both feet was 10. In the very rare patient with an amputation, we assessed the remaining foot and doubled this to estimate the MNSI. DPN was defined as a MNSI score >2.0 [17,20].

The peripheral vascular examination included palpation of the dorsalis pedis and posterior tibial pulses, and each pulse was defined as present or absent. If all four pedal pulses were palpable, no further evaluation took place unless the patient had previously undergone revascularization of the lower extremity. Patients with an abnormal examination were sent for noninvasive arterial studies. PAD was defined as an ankle–brachial index (ABI) <0.9. Those patients whose ABI was >1.3 were also diagnosed to have PAD if the pulse volume recording waveforms were dampened [21].

The data were analyzed using the Pearson chi 2 tests. Univariate logistic regression was used to analyze dichotomous variables and Student *t* tests with equal variance were used to determine significant differences of the means of normally distributed continuous variables (i.e. age) between groups. Statistical significance was set at *p*< 0.05.

## Results

The demographics of the study and control groups are listed in Table 1. Diabetic patients with and without CN were similar in all measured variables with the exception of peripheral neuropathy. Although our non-Charcot cohort had a high prevalence of neuropathy (78%) this was significantly lower than the patients with CN (p = 0.003). The mean preoperative serum 25-OH vitamin D3 values in patients with (20.3 ng/mL) and without CN (21.6 ng/mL) was not significantly different (p = 0.55) (Table 1). This ostensible similarity between groups is likely due to the high prevalence of DPN (90%) in the entire cohort of diabetic patients. Nonetheless, both groups of patients demonstrated levels approaching Vitamin D deficiency.

**Table 1.** Charcot versus non-charcot.

	Non-CN n (%)	CN n (%)	p value
n	50	50	
Age	55.7	55.9	0.95
Males: Females	28 (56): 22 (44)	28 (56): 22 (44)	1
Type I: Type II	9 (18): 41 (82)	9 (9): 41 (82)	1
PAD	19 (38)	8 (16)	0.84
Neuropathy	39 (78)	50 (100)	0.003
Ulceration	25 (50)	29 (58)	0.42
Infection	13 (26)	20 (40)	0.22
Smoking	13 (26)	9 (18)	0.34
Transplant	3 (6)	8 (16)	0.11
BMI	30.9	33.8	0.08
Glucose	167 mg/dL	175 mg/dL	0.62
HgA1C	7.7%	7.7%	0.89
Creatinine	1.7 mL/min	1.5 mL/min	0.70
Vit D3	21.6 ng/mL	20.3 ng/mL	0.55
Albumin	3.4 g/mL	3.5 g/mL	0.78

Demographics and laboratory values for patients with and without Charcot neuropathy are noted.

The secondary evaluation was performed using the various diabetic foot complications including PAD, DFI, DPN, and DFU. The demographics of PAD comparative groups are listed on Table 2. Diabetic patients with PAD were more likely to be males (p = 0.009) who used tobacco (p = 0.001). Patients with PAD had a higher rate of active infections than patients without PAD (p = 0.003). Diabetic patients with PAD had lower levels of vitamin D3 (p = 0.03) and serum albumin (p = 0.02), as well as higher serum creatinine levels (p = 0.007) than diabetic patients without PAD. Although there was a trend towards higher rates of DPN and DFU in patients with PAD compared to patients without PAD, this difference did not reach statistical significance in our cohort (p = 0.07).

**Table 2.** Peripheral arterial disease versus non-peripheral arterial disease.

	Non-PAD n (%)	PAD n (%)	p value
n	79	22	
Age	55.6	58.5	0.22
Males: Females	39(49): 40 (51)	17 (77): 5 (23)	0.009
Type 1: Type 2	15(19): 64 (81)	3(14): 18 (86)	0.62
Neuropathy	68 (86)	21 (95)	0.07
Ulceration	39 (49)	15 (68)	0.07
Infection	25 (32)	14 (64)	0.003
CN	42 (53)	8 (36)	0.22
Smoking	12 (15)	19 (86)	0.001
Transplant	9 (11)	2 (9)	0.81
BMI	33.0	30.2	0.16
Glucose	169 mg/dL	179 mg/dL	0.63
HgA1C	7.8%	7.5%	0.49
Creatinine	1.4 mL/min	2.4 mL/min	0.007
Vit D3	22.2 ng/mL	16.2 ng/mL	0.03
Albumin	3.7 g/mL	2.9 g/mL	0.02

Demographics and laboratory values for patients with and without peripheral arterial disease are noted.

Thirty-nine percent of the patients in this study had a DFI and the comparisons between non-infected patients are listed in Table 3. Patients with DFI were more likely to be males (p = 0.03) with PAD (p = 0.003), DPN (p = 0.005), and DFU (p < 0.0001). Patients with DFI had lower levels of vitamin D3 (p = 0.0006), lower serum albumin (p = 0.001) and higher creatinine levels (p < 0.0001) than patients without DFI.

**Table 3.** Infected versus non-infected.

	Non-Infected n (%)	Infected n (%)	p value
n	61	39	
Age	56.2	55.1	0.64
Males: Females	29 (48): 32 (52)	27 (69): 12 (31)	0.03
Type 1: Type 2	12 (20): 49 (80)	6 (15): 33 (85)	0.59
PAD	7 (11)	14 (36)	0.003
Neuropathy	50 (82)	39 (100)	0.005
Ulceration	18 (30)	36 (92)	<0.0001
CN	30 (49)	20 (51)	0.84
Smoking	13 (21)	9 (23)	0.84
Transplant	7 (11)	4 (10)	0.85
BMI	31.8	33.3	0.39
Glucose	163 mg/dL	184 mg/dL	0.23
HgA1C	7.7%	7.7%	0.81
Creatinine	1.1 mL/min	2.4 mL/min	<0.0001
Vit D3	23.9 ng/mL	16.3 ng/mL	0.0006
Albumin	4.0 g/mL	2.8 g/mL	0.001

Demographics and laboratory values for patients with and without diabetic foot infection are noted.

Eighty-nine percent of the patients in this study had DPN and the demographics of those with and without DPN are listed on Table 4. Comparisons are limited due to the fact that all diabetic foot complications (PAD, CN, DFU, etc.) were reported only in the neuropathic group. There was a strong trend towards lower levels of vitamin D3 (p = 0.05) in patients with neuropathy compared to patients without neuropathy (p = 0.05). Conversely, serum creatinine levels were higher in patients with peripheral neuropathy compared to those without neuropathy (p = 0.0003).

**Table 4.** Neuropathic versus non-neuropathic.

	Non-Neuropathic n (%)	Neuropathic n (%)	p value
n	11	89	
Age	52.7	56.2	0.37
Males: Females	7 (64):4 (36)	37 (42):52 (58)	0.2
Type 1: Type 2	0 (0):11 (100)	18 (20):71 (80)	N/A
PAD	0 (0)	21 (24)	N/A
Infected	0 (0)	39 (44)	N/A
Ulceration	0 (0)	54 (61)	N/A
CN	0 (0)	50 (56)	N/A
Smoking	4 (36)	18 (20)	0.33
Transplant	0 (0)	11 (12)	N/A
BMI	32	32.4	0.85
Glucose	141 mg/dL	175 mg/dL	0.13
HgA1C	6.9%	7.8%	0.13
Creatinine	0.97 mL/min	1.68 mL/min	0.0003
Vit D3	27 ng/mL	20.2 ng/mL	0.05
Albumin	4.0 g/mL	3.5 g/mL	0.06

Demographics and laboratory values for patients with and without diabetic neuropathy are noted.

Patients were tracked for both a current DFU (n = 54) and a history of DFU (n = 62). To ensure assess the relevance of serum vitamin D3 level and DFU, only patients with a current DFU were evaluated. Fifty-four percent of the patients in this study had a current DFU at the time of surgery (Table 5). Patients with a DFU were more likely to be males (p > 0.001) with DPN (p < 0.001), DFI (p < 0.001), lower levels of vitamin D3 (p = 0.03), lower serum albumin (p = 0.004), and higher creatinine levels (p = 0.04) than patients without a current of DFU. Trends were noted between DFU and a history of transplantation (p = 0.05) and PAD (p = 0.07).

**Table 5.** Current ulcer versus no current ulcer.

	No ulcer n (%)	Ulcer n (%)	p value
n	46	54	
Age	55.5	56.1	0.79
Males: Females	17 (37):29 (63)	39(72):15 (28)	>0.001
Type 1: Type 2	7 (15):39 (85)	11 (20):43 (80)	0.50
PAD	6 (13)	15 (28)	0.07
Infected	3 (7)	36 (67)	>0.001
Neuropathy	35 (76)	54 (100)	>0.001
CN	21 (46)	29 (54)	0.42
Smoking	12 (26)	10 (19)	0.36
Transplant	2 (4)	9 (17)	0.05
BMI	32.6	32.2	0.84
Glucose	159 mg/dL	182 mg/dL	0.18
HgA1C	7.5%	7.9%	0.33
Creatinine	1.2 mL/min	1.9 mL/min	0.04
Vit D3	23.6 ng/mL	18.7 ng/mL	0.03
Albumin	4.0 g/mL	3.0 g/mL	0.004

Demographics and laboratory values for patients with and without diabetic foot ulceration are noted.

In our overall cohort of 100 patients, the mean serum vitamin D3 level was 20.96 ng/mL, a value which falls into the category of vitamin D insufficiency. Fifty-five percent of the patients were found to be vitamin D deficient, 24% were found to be vitamin D insufficient, and 21 had adequate serum vitamin D3 levels (Table 6). The effect of seasonal fluctuation was also evaluated and reported in Figure 1. While the lowest serum vitamin D average was noted in the spring, no statistically significant difference was noted between all four seasons.

**Table 6.** Serum vitamin D3 levels.

	n	Mean ng/mL
Vitamin D deficient^#^	55	13
Vitamin D insufficient*	24	25
Vitamin D adequate^^^	21	37

#Serum vitamin D3 < 21 ng/mL *Serum vitamin D3 21–29 ng/mL ^Serum vitamin D3 > 28 ng/mL

**Figure 1.**
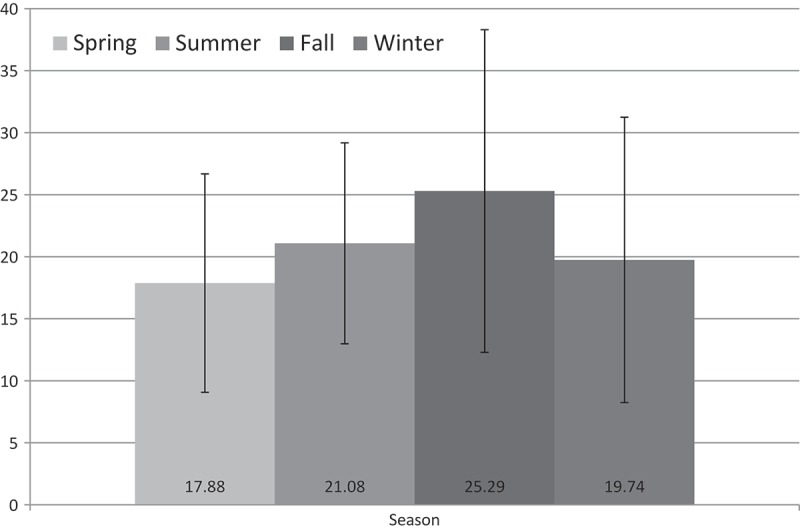
Seasonal comparison. While season fluctuation was noted, no significant difference was noted between the four seasons.

## Discussion

Vitamin D is derived from two main sources. Endogenous production from skin exposure to direct sunlight and exogenous dietary sources such as fish, dairy, and supplements [6]. Endogenous production is far more effective in binding to carrier proteins and increasing serum vitamin D levels. A fair skinned individual will produce about 20,000 IU of vitamin D from 30 min of sun exposure. Individuals with darker complexion require 20–30 times more sun exposure to receive the same effect. Once pre-vitamin D is produced or absorbed, hydroxylation occurs in the liver forming 25-vitamin D3 [6]. A second hydroxylation must occur for the production of the active form [6]. This occurs mainly in the kidney but also occurs in other cells throughout the body. Since many patients with diabetes-related foot complications have impaired renal function, it is not surprising that such a high percentage of our patients had reduced levels of serum vitamin D.

Monitoring vitamin D levels is an important for foot and ankle surgeons for a number of reasons. Serum vitamin D levels have been shown to improve glycemic control in patients with DM [7,22,23]. Activated vitamin D is necessary for proper insulin receptor function and has been associated with the development of mellitus [6]. Kant et al. evaluated vitamin D levels in patients with Type 2 DM and found that 91% of patients had some form of vitamin D deficiency [7]. The frequency of vitamin D deficiency in patients with DM is unknown.

A number of diabetic complications have been associated with low serum vitamin D levels, including cardiovascular disease and arterial wall stiffness. Higher ankle-brachial indices (>1.3) were found in patients with lower vitamin D levels [11]. A meta-analysis performed by Iannuzzo et al. found that PAD patients have lower vitamin D levels than controls and both vitamin D deficiency and vitamin D insufficiency are significantly associated with PAD [9]. Melamed et al. reported that vitamin D levels were independently associated with PAD; for each decrease of 10 ng/mL in the 25-hydroxyvitamin D level, the multivariable-adjusted prevalence ratio of PAD was 1.35 (95% confidence interval, 1.15–1.59) [10]. Reis et al. postulated that the racial differences in vitamin D concentrations could explain nearly one-third of the excess risk of PAD above and beyond differences in established and novel risk factors for cardiovascular disease in black adults [24]. The risk of PAD may be decreased with vitamin D supplementation, potentially due to several different mechanisms. First, vitamin D has been shown to play a vital role in the renin-angiotensin-aldosterone system pathway [25]. Therefore, higher levels of serum vitamin D may lower blood pressure. Second, vitamin D improves endothelial functioning. Sugden et al. found that a single high-dose of vitamin D2 increases flow-mediated vasodilatation of the brachial artery by 2.3% [26]. In patients with chronic kidney disease, Garcia–Canton et al. found that vitamin D levels and vascular calcification scores were inversely related [27].

The effects of serum vitamin D and DPN are also well documented. A meta-analysis performed by Qu et al. found Asian type 2 diabetic patients with vitamin D deficiency are 1.22 times to suffer from DPN compared with normal vitamin D levels [28]. Shehab et al. also found DPN was significantly associated with vitamin D deficiency (p = 0.04) after inclusion of potential confounders such as duration of diabetes, HbA1c and LDL-cholesterol [29]. Basit found that a single high-dose intramuscular injection of vitamin D3 in patients with painful DPN was associated with a significant decrease in the symptoms (p < 0.001) [30]. The authors then presented a follow-up study demonstrating improved quality of life in patients with painful DPN treated with the single high-dose intramuscular injection of vitamin D3 [31].

Emerging data also appears to demonstrate a negative relationship between DFU and low serum vitamin D. Feldkamp et al. also showed a negative correlation (r = −0.241) (p < 00.1) between severity of a DFU, using the UT ulcer classification [32], and 25-hydroxyvitamin D3 status [14]. Our findings strongly correlate with Feldkamp et al., demonstrating that low serum vitamin D levels are associated with increased risk of DFI, PAD, and DFU.

Patients with complications of DM (DPN, PAD and renal disease) who undergo foot and ankle surgery are at increased risk of perioperative complications [33]. These adverse outcomes include surgical site infection, wound healing problems, nonunion and hardware failure. Vitamin D has been shown to be an immunostimulant, and is a necessary component in the activation of T and B immune cells by macrophages [34]. Jeng et al. found vitamin D binding protein levels in plasma were significantly lower in critically ill subjects with sepsis compared to critically ill subjects without sepsis [35]. They also found a positive association between serum vitamin D and response by induction of cathelicidin; which may indicate that optimal vitamin D status may be important for innate immunity, especially in the setting of sepsis [35]. Vitamin D has been shown to increase the ability of keratinocytes to kill *Staphylococcus aureus* [36] and has also been shown to play a role in nasal carriers of methicillin-resistant *Staphylococcus aureus* (MRSA) [37]. Vitamin D supplementation may also improve healing by decreasing chronic inflammation markers including C reactive protein, tumor necrosis α, a lower erythrocyte sedimentation rate, and higher levels of leptin [12,13]. Given the significant associations between low vitamin D and PAD and DFI, normalization of vitamin D levels potentially can improve patient health and therefore decrease these risks.

Vitamin D plays a well-known and important role in optimizing bone health by maintaining calcium homeostasis, largely secondary enhancing intestinal absorption of calcium [6]. In patients with vitamin D deficiency, serum hypocalcemia stimulates parathyroid hormone production. This secondary hyperparathyroidism leads to increased osteoclastic activity and resorption of bone to maintain serum calcium levels at physiologic levels. This mineral leaching leads to reduction in bone mineral density, osteopenia and osteoporosis. Patients with DPN are at particular risk for nonunion, malunion, hardware failure and pathologic fractures due to impaired bone health [38,39]. Due to profound DPN, these patients often have balance issues and increased risk of falls. Both of these can place increased stress on fixation devices (internal and external), and coupled with reduced bone quality, may contribute for adverse outcomes. Because of the DPN induced balance issues and fall risk, our patients often are not compliant with non-weight bearing instructions. While others have suggested that vitamin D deficiency may play a role in the development of CN [8], we were unable to identify a significant difference in diabetic patients with and without CN. This may be related to the high prevalence of DPN in the non-Charcot cohort (78%).

Our study has a number of limitations. Retrospective studies rely on the accuracy of recorded data, and therefore, are prone to potential errors in data collection. The introduction of matching in this study, while based on age, gender, and type of diabetes, also introduces the possibility of bias. Due to the geographic location of the study (Pittsburgh, PA), a selection bias may be present. Latitude north of the 37^th^ parallel has a known effect of serum vitamin D due to reduce production, especially during the winter months. Our study did not find a statistical difference based on season, reducing the risk of seasonal selective bias. We also note that our groups for secondary outcomes have relatively small numbers. Due to the presence of smaller group sizes, there is a risk of selection bias and possible over or underestimation of the effect of the variable due to inadequate power to detect any real differences. Another limitation of this study is the cohort of patients studied. Our practice is primarily a tertiary care academic referral center, and this potentially introduces further selection bias into our study and control groups. Eighty-nine percent of patients had neuropathy, a comorbidity that is associated with diabetes-related foot complications (DFI, DFU, CN, and PAD). Consequently, the results of this study may not be generalizable to the general diabetic population who do not have foot-related complications. Due to the high level of neuropathy in both the patients with and without CN, this has limited our understanding of the effect that neuropathy may have on the study outcomes. A final limitation is that we do not assess if supplementation can reduce risk or prevent these complications from occurring. Our study is only noting the increased prevalence of low serum vitamin D3 in this select population of diabetic subjects.

## Conclusion

Vitamin D deficiency is a common but potentially treatable condition. Seventy-nine percent of the patients in this study had either vitamin D deficiency or insufficiency. In this study we found that vitamin D deficiency is associated with peripheral arterial disease (PAD), infection (DFI), peripheral neuropathy (DPN), and current ulceration (DFU). Patients suffering from Charcot neuroarthropathy (CN) did not have lower serum vitamin D levels than our diabetic patients with no history of CN although both groups demonstrated an insufficiency. Surgeons should be aware of the increased likelihood of vitamin D deficiency and insufficiency in patients with complications of diabetes mellitus such as PAD, DFI, DPN, and DFU. Surgeons should consider measuring vitamin D levels in high-risk patients in an effort to identify deficiency and insufficiency. This includes not only patients with DM, but also pregnant or lactating mothers, elderly, or those who live above the 37th parallel. Although foot and ankle surgeons are largely concerned with the distal lower extremity, identification of vitamin D deficiency/insufficiency can identify patients at high risk for life threatening fractures of the spine, pelvis or hip. Oral supplementation with vitamin D can be instituted or referral to the appropriate medical specialist can be made.
